# Involving Health Care Professionals in the Human-Centered Design of a Digital Platform for Work-Focused Health Care: Lessons From a Mixed Methods Study

**DOI:** 10.2196/83212

**Published:** 2026-04-17

**Authors:** Nina Zipfel, Marije Hagendijk, Ersen Colkesen, Marijke Melles, Sylvia J van der Burg-Vermeulen

**Affiliations:** 1 Amsterdam UMC location University of Amsterdam, Department of Public and Occupational Health, Coronel Institute of Occupational Health Amsterdam The Netherlands; 2 Amsterdam Public Health Research Institute, Societal Participation & Health Amsterdam The Netherlands; 3 Department of Cardiology St. Antonius Hospital Nieuwegein The Netherlands; 4 Faculty of Industrial Design Engineering Delft University of Technology Delft The Netherlands

**Keywords:** human-centered design, work-focused health care, networked-care platforms, end-user involvement, co-creation, digital platform development

## Abstract

**Background:**

Effective collaboration throughout the full cycle of care is essential for value-based health care. In the Netherlands, occupational health care and curative health care traditionally operate as 2 separate sectors. As a consequence, effective communication and robust collaboration between professionals working in these sectors are lacking. Digital collaborative care platforms (ie, digital systems that facilitate communication and collaboration between health care professionals) are recognized as a promising solution to address the fragmentation of work-focused health care (health care that supports people on long-term sick leave in staying at or returning to work). A human-centered design (HCD) approach can help ensure that such platforms align with professionals’ needs by involving them throughout the design process.

**Objective:**

This study examines the experiences of (work-focused) health care professionals, including occupational physicians, insurance physicians, medical specialists, and general practitioners, during the design phase of a real-world HCD process for developing a digital platform to support collaborative care. The study specifically focused on understanding how these professionals perceive this collaborative approach.

**Methods:**

A mixed method study design was employed, combining observations of 17 design sessions with semistructured interviews with health care professionals as intended users of the platform. Observational data captured session dynamics, while interview data provided deeper insights into professionals’ experiences with the participatory HCD approach.

**Results:**

Health care professionals were generally motivated to contribute, driven by professional interest, social encouragement, or a desire to improve practice. They valued the open and informal atmosphere of the design sessions and described their role as actively sharing practical experiences and identifying bottlenecks in current practice. Participants emphasized the importance of clear goals, good preparation, and iterative involvement for meaningful engagement. Barriers identified included limited session time, constraints of virtual interaction, and uncertainty about the commercial context of the platform. Some professionals felt unsure about the relevance of their input or experienced limited interaction, especially when the session’s purpose was unclear. Others noted that the use of a mock-up platform as a conversational foundation, familiarity with similar system interfaces, and well-guided, structured discussions facilitated their input. Positive experiences included a sense of impact through involvement in the design process, note-taking as part of active user engagement, and a safe environment for open and constructive feedback. Participants recommended a clearer explanation of the platform’s broader aims in advance, enhanced participant preparation, and opportunities for multidisciplinary co-creation in future sessions.

**Conclusions:**

Health care professionals valued being part of the collaborative design process, but their engagement and perceived contribution were highly dependent on how the design sessions were facilitated. Structuring design sessions with clear expectations, preparatory tools, and opportunities for follow-up can support more effective, foundational co-creation in digital platform development for collaboration among professionals providing work-focused health care.

## Introduction

A major challenge in health care is the fragmentation between work-focused health care and curative health care. In the Netherlands, these sectors function as 2 separate entities that collaborate suboptimally [1,2]. Work-focused health care refers to the system and professionals, including occupational physicians, insurance physicians, and labor experts, who are responsible for work-focused health guidance, return-to-work (RTW) support, and work disability assessment, as regulated by the Dutch Gatekeeper Improvement Act and the social insurance system. Curative health care refers to medical care provided by general practitioners and medical specialists, aimed at diagnosing, treating, and curing diseases. Organizational and operational structures for direct referral, reimbursement, and multidisciplinary consultation and collaboration are insufficient or lacking [3,4]. Furthermore, communication between professionals operating within these 2 distinct health care entities is inadequate and inefficient [5]. This lack of coordination has significant implications for work-focused health care, particularly in addressing key challenges such as an aging workforce and the increasing prevalence of chronic diseases within the working population [6]. Inefficient collaboration between occupational and curative health care can lead to delays in RTW processes, increased administrative burden, and suboptimal support for employees with complex or long-term health conditions [6,7]. Therefore, there is a clear need for a digital infrastructure to enhance communication and collaboration between the work-focused and curative care sectors [5]. Digital platforms have the potential to directly address these inefficiencies by facilitating real-time information exchange, enabling seamless multidisciplinary consultation, and integrating care pathways across occupational and curative health care sectors [8]. By breaking down silos and supporting coordinated action, such platforms can reduce administrative burden, minimize delays in RTW processes, and improve outcomes for workers with complex or long-term health conditions [2,9].

A promising strategy to address these problems is to establish regional work-focused collaborative care networks in which all relevant health care professionals—from both work-focused and curative care (ie, medical specialists, general practitioners, occupational health physicians, and insurance physicians)—work together throughout the full cycle of care surrounding the sick worker [5]. Such collaborative care networks could contribute to sustainable employability. To operate effectively and efficiently, these networks should ideally be supported by digital platforms for information sharing, care planning, and digital multidisciplinary consultation [5]. Although regional collaborative care via digital platforms is endorsed by governmental authorities [10] in the Netherlands, insights into the design and implementation of such platforms across sectors are still lacking. To ensure sustainable implementation and use, it is highly important to align the platform with the functional and technical requirements of all users and stakeholders involved [11].

In this context, human-centered design (HCD) has gained prominence as an essential approach for integrated health care innovations, as it helps ensure that interventions are tailored to the real needs of patients, caregivers, and health care providers [11]. HCD is a person-focused methodology that aims to create intuitive, effective, and meaningful interventions by involving key professionals throughout the development process [11,12]. While user-centered design focuses on optimizing usability for end users, HCD adopts a broader, more holistic approach that considers the needs, values, and context of all people involved, including professionals, patients, and other stakeholders. The goal of HCD is to align human needs with complex environments such as work-focused care and curative care [11,13,14]. A fundamental principle of HCD is the initial assessment of the target population’s needs, ensuring that solutions are tailored to the intended users [11,15]. A common HCD approach is the 4-phased double-diamond approach—(1) discover, (2) define, (3) design, and (4) validate—which enables iterative development through continuous stakeholder engagement [11,16]. The (1) discover phase aims to comprehensively understand the problem, informing the (2) define phase to clarify the actual problem. The (3) design phase employs participatory methods, such as co-creation, to address the defined problem. Finally, in the (4) validate phase, small-scale testing is conducted on different interventions to identify the most suitable one. These phases are often framed in a double-diamond model to reflect the iterative nature of exploring various solution options before narrowing down to the most suitable solution [11]. HCD adopts a holistic approach, considering a system of interacting and interdependent stakeholders rather than a single group [11]. In health care, HCD is widely applied in health technology innovation [14], especially in integrated care where multiprofessional collaboration is essential. Health challenges such as chronic diseases, aging populations, and mental health issues require multistakeholder input from patients, care providers, policy makers, and IT developers to co-create context-aware innovations that enhance usability and practical implementation [17]. Additionally, health care technologies often face resistance due to usability or workflow issues [8], but involving end users in co-design fosters engagement, co-ownership, acceptance, and trust [8]. In this way, HCD-driven development helps address implementation challenges, especially in networked care models that rely on interoperability, data sharing, and coordinated workflows [11].

This study explores how health care professionals perceive their involvement and the value they attribute to participating in the design of a platform aimed at enhancing interprofessional collaboration to facilitate a collaborative care network for work-focused health care. By addressing these objectives, the study provides insights into the practical use of collaborative design sessions in health care technology development and offers recommendations to optimize user involvement in the design and implementation of digital platforms that support collaboration between work-focused health care and curative care.

## Methods

### Study Design and Setting

The study used a mixed method design, combining qualitative observations of design sessions with semistructured in-depth interviews to gain insight into the experiences of health care professionals as intended users of the platform. Observations were used to inform and tailor the subsequent interviews. This study adhered to the COREQ (Consolidated Criteria for Reporting Qualitative Research) checklist to ensure comprehensive and transparent reporting of qualitative research methods and findings. The completed COREQ checklist is provided in Multimedia Appendix 1.

**Figure 1:**
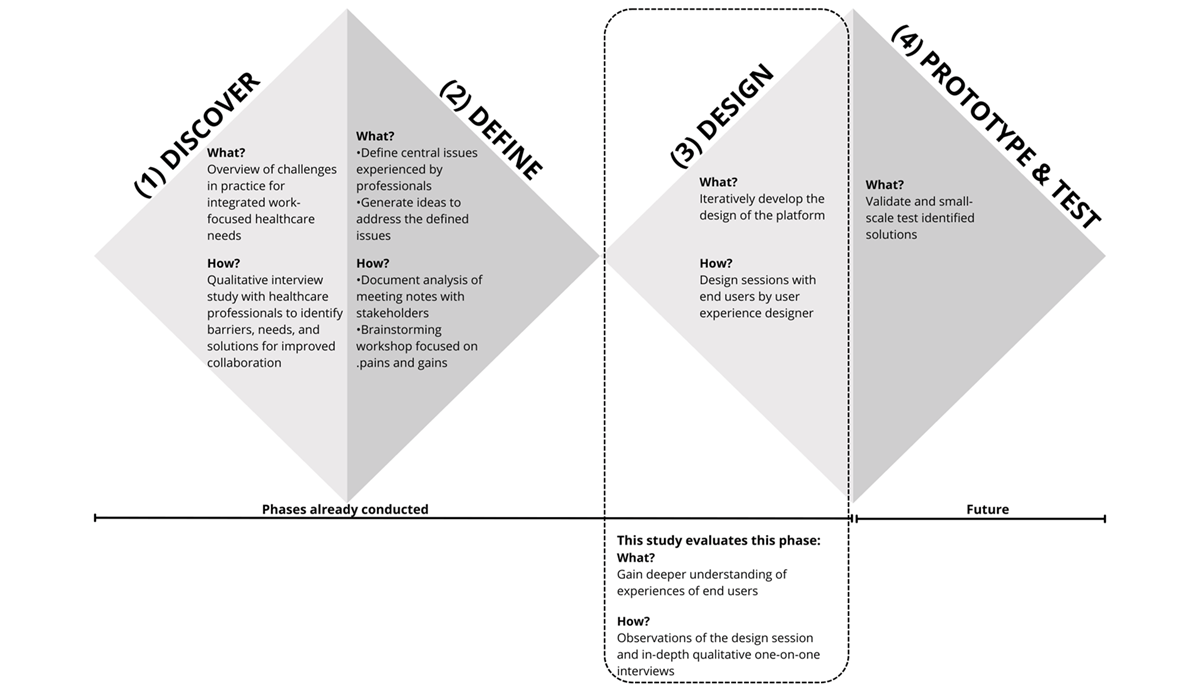
Overview of the person-centered design approach and the focus of the current mixed-methods study evaluating experiences of healthcare professional during the design phase of a digital platform for work-focused healthcare in the Netherlands based on Melles et al. [10, 15].

This study specifically evaluates (work-focused) health care professionals’ experiences during a design session in the development of a digital platform, as part of a broader design process (Figure 1). While design processes using an HCD approach may involve a diverse range of stakeholders, including decision makers, policy makers, and insurers, this study focuses exclusively on health care professionals, aiming to gain a deeper understanding of their experiences and to ensure the platform’s relevance to their daily practices. The first 2 phases, discover and define, were part of earlier studies that examined both patients’ and professionals’ perspectives [1,2,18]. In the discover phase, qualitative interviews and patient journey mapping were used to explore the experiences, needs, and barriers of workers living with cardiovascular disease, as well as the perspectives of health care professionals involved in work-focused health care [1,2]. Key findings from these studies highlighted persistent fragmentation and suboptimal communication between occupational and curative health care, insufficient multidisciplinary collaboration, and a lack of digital infrastructure to support RTW processes. Both patients and professionals expressed a need for better information exchange, clearer role definitions, and more integrated care pathways [1,2,18]. The involvement of people with lived experience (patients and professionals) was central to these phases, ensuring that the design constraints and priorities reflected real-world practice. The detailed results of these phases are published elsewhere [1,2,18]. The study setting was embedded within the Value@WORK research project, which focuses on implementing value-based health care and promoting collaboration in a work-focused collaborative care network for patients with cardiovascular disease who experience work-related problems.

### Phase (3) Design: Setup Design Sessions

The design sessions were led by a company specializing in the development of digital infrastructure for health care professionals, specifically a digital platform that supports interprofessional communication and collaboration among professionals [19]. A user experience (UX) designer from this company, who was responsible for developing the digital platform, facilitated the sessions. In these one-on-one sessions, the designer presented mock-up versions of a potential platform (ie, a static representation of the envisioned digital interface) and invited health care professionals to provide feedback. In addition, a platform developer was present in each session to explain the platform’s goals and to ensure maximal learning. The researchers were involved as independent observers, capturing the experiences of health care professionals as intended users of the platform throughout the process. To support an unbiased evaluation, clear agreements were established regarding data governance, confidentiality, and independent analysis of the findings. The researchers had full autonomy in conducting the evaluation and interpreting the data, separate from the platform developers. The design session format (see also Table 1) followed these steps: (1) an introduction to the broader context and purpose of the platform, (2) presentation of a mock-up version of the platform, (3) an interactive discussion with the participant, and (4) a general wrap-up of key insights and next steps. All design sessions were held online via Microsoft Teams and lasted approximately 1 hour. The sessions were conducted using the same design, which did not change during this phase. Although the design sessions were intended as co-design activities, in practice they often focused on idea generation and feedback on a mock-up rather than fully participatory co-creation. This distinction is important for interpreting the level of user involvement and the rigor of the HCD process in this study.

**Table 1:** Format of the one-on-one design session conducted with 17 health care professionals as part of a mixed methods study on the development of a digital platform for work-focused health care in the Netherlands.

Step in the design session format	Explanation of this step
1. Introduction	A typical session began with the platform developers providing an overview of the broader context of integrated work-focused health care and the platform’s purpose. The UX^a^ designer then explained the process and set the framework for the design sessions. Depending on the participant’s familiarity with the goals of the design, this step ranged from brief to more detailed.
2. Presentation of the platform mock-up	The UX designer shared their screen via Microsoft Teams to present a static mock-up of the platform. Key features, such as a timeline, document-sharing tools, agenda functionalities, and user permissions, were demonstrated. During this step, participants observed but did not interact directly with the mock-up.
3. Interactive discussion with participants	During this step, open, high-level questions invited participants to share their thoughts on the platform’s overall design, usability, and alignment with their professional needs. The UX designer clarified participants’ questions and elaborated on specific features of the mock-up version of the platform. The platform developers provided additional context, particularly in areas related to business considerations, finance, and future implementation. The discussion focused on general impressions, perceived usefulness, and potential barriers or facilitators for implementation rather than on detailed workflow or task-based usability testing. Participants did not interact directly with the mock-up; instead, they provided feedback based on the demonstration and their professional experience. Discussions often addressed workflow inefficiencies, privacy concerns, and role-specific requirements, ensuring that the feedback was relevant and actionable.
4. General wrap-up	The design sessions concluded with a summary of the key points discussed and requests for further participation in future iterations of the platform.

^a^UX: user experience.

### Participants and Recruitment

The study included 17 health care professionals who each participated in an individual design session as intended users of the platform under development. Each participant was involved in a single design session. These participants represented both work-focused health care professionals (ie, occupational health physicians and social insurance physicians) and curative care professionals (ie, medical specialists and general practitioners). Participants took part in the design sessions, as described above, and were also included in the evaluation of the HCD process. This ensured that all professionals who engaged with the mock-up during the (3) design phase also provided feedback on their experiences.

The study involved health care professionals for whom the new platform is intended to be used, including 3 insurance physicians working with the Dutch social security agency, 1 policy advisor from the same agency with a focus on digital technology development, 1 reintegration counselor from the Dutch social security agency, 1 private insurance physician, 5 occupational physicians, 2 cardiologists, 2 general practitioners, 1 physiotherapist, and 1 clinical psychologist. These professionals were selected because they are involved in providing both work-focused and curative care, as identified by patients in an earlier study [1]. Participants were recruited using a combination of convenience and snowball sampling from the networks of the researchers and the developers. While the platform developers led the organization of the design sessions, recruitment was a shared effort based on a jointly defined overview of professionals involved in work-focused health care, informed by earlier studies [1,2]. Notably, the evaluation of the experiences of professionals intended to use the platform was initiated independently by the researchers and agreed upon in advance. All health care professionals who participated in a design session were also invited and agreed to take part in the interviews for the purpose of this study.

### Data Collection and Analysis

Data were collected from (1) observations of the one-on-one design sessions and (2) one-on-one semistructured interviews with participants after their participation in the design session. All data were collected between June and September 2023. Both the design sessions and the one-on-one interviews were conducted online via Microsoft Teams. The design sessions were observed by the first author (NZ), who also conducted the one-on-one interviews.

### Observations

As part of the data collection process, all design sessions conducted in phase 3 were systematically observed using a structured observation checklist to gain deeper insights into UX with the participatory HCD approach during platform development. This checklist was developed for the study and included predefined categories related to session structure, participant and UX designer behavior, and the use of supporting materials. Particular attention was given to nonverbal cues, hesitations, and spontaneous feedback, providing a deeper understanding of how professionals experienced their involvement in the design session. The approach was primarily deductive, but notes were also taken on any additional relevant behaviors or events that emerged during the sessions. The detailed observation checklist is presented in Multimedia Appendix 2.

The insights from these observations were used to structure and refine the subsequent semistructured interviews, ensuring that questions addressed key aspects of the collaborative approach during the design session, such as users’ perceptions of collaboration, their sense of influence over design decisions, and their overall engagement in the process. By identifying patterns in user responses and areas of uncertainty or enthusiasm, the observations helped guide a more in-depth exploration of health care professionals’ experiences, leading to a richer understanding of the impact of the participatory HCD approach. The observational data were primarily used to contextualize the results and to inform the development and focus of the subsequent qualitative interviews, thereby enhancing the relevance and depth of the insights gathered.

### Semistructured Interviews

To gain a deeper understanding of the experiences of the involved health care professionals, qualitative semistructured interviews were conducted with all participants following the design session. An interview guide was developed to gain insights into participants’ motivations for joining the session and their experiences. It included questions about their expectations for participating in the design session, their overall experience, their perceived role, the extent to which they felt prepared, their experience with the materials used, their communication with the UX designers, and their envisioned future role after participating in the design session. The interview guide served as a memory aid during the interviews. The detailed interview guide is presented in Multimedia Appendix 3. Interviews were conducted individually following the design session and lasted approximately 30 minutes. All interviews were audio-recorded with participants’ consent and later transcribed verbatim. The data were then analyzed using thematic analysis, which involved coding the transcripts to identify key themes and patterns. Thematic analysis of the interview data was primarily inductive, with codes and themes developed from the data. However, the structuring and interpretation of themes were guided by the “4 E’s of HCD participation” framework, providing a deductive lens for organizing the findings [12]. This method allowed for a systematic analysis of the structure, process, experiences, perceptions, and feedback shared by participants [12]. To guide the structuring and interpretation of themes, the “4 E’s of HCD participation” framework was used: *engage, elicit, elaborate,* and *evaluate* [12]. *Engage* refers to the process of actively involving participants in the design process, ensuring they are invested in and committed to the development of the platform. *Elicit* involves gathering input from participants to understand their needs, challenges, and expectations regarding the platform. *Elaborate* entails refining and expanding on the gathered input, allowing for deeper discussion and clarification of participants’ perspectives. *Evaluate* focuses on assessing the effectiveness of the design, ensuring that the platform aligns with users’ needs and improves their work-focused practices. All data were analyzed in MAXQDA.

### Role of the Researchers

The first (NZ) and second (MEH) authors are full-time researchers with demonstrated expertise in qualitative research methods. The fourth author (MMM), in addition to being a full-time researcher with expertise in qualitative research methods, provided expertise in HCD and user involvement in the development process. The other authors (BEC and SJvdBV) are experienced researchers with knowledge of both the curative care sector and the work-focused care sector, contributing to the relevance of this study. Notably, none of the authors were involved in the design of the design sessions. While this independent position may limit the ability to fully assess certain aspects of the design process, it also allowed for an unbiased observation and evaluation of participant experiences. However, it may have limited the ability to fully capture the rationale behind certain design choices or the nuances of the facilitation process.

### Ethical Considerations

This study was reviewed by the Medical Ethics Committee of the Amsterdam University Medical Center, which determined that it did not fall under the scope of the Medical Research Involving Human Subjects Act (Reference 2023.0463). Therefore, formal ethics approval was not required according to institutional and national guidelines. All participants provided informed consent for the researcher to observe the sessions and conduct interviews before participation. Participation was voluntary, and participants could withdraw at any time without consequence. To ensure privacy and confidentiality, all data were anonymized and deidentified before analysis. Audio recordings were transcribed verbatim, and any identifying information was removed. Data were stored securely and were accessible only to the research team. No compensation was provided to participants for their involvement in the study.

## Results

### Overview

In total, 17 individual one-on-one sessions focusing on the same design were conducted, each followed by a one-on-one interview to explore participants’ experiences with the session. The section below presents the findings, structured along the 4 E’s of HCD participation: *engage, elicit, elaborate,* and *evaluate* [12].

### Engage: Motivations and Preconditions for Participation

Participants described various reasons that shaped their motivation for joining the design sessions. These motivations reflected both intrinsic professional drivers and contextual conditions that enabled or constrained engagement. Some professionals indicated that they were driven by a desire to “contribute to improving the quality of the profession,” aiming to enhance their field of practice. Others appreciated that “participation provides variety in work” and viewed the design sessions as a welcome change from routine tasks. Additionally, professionals reported being “motivated for change,” particularly in addressing long-standing communication barriers. Curiosity also played a role, especially among those with “prior exposure to digital innovations” or a “professional expertise and interest in technology.” As one insurance physician explained:

...through my work, I deal with aspects like medical confidentiality and GDPR. Data sharing is part of my portfolio, and I am also involved in the IT side of things. I am not an IT professional, but whenever an IT system is being developed, they involve me. So, for that reason, I do have some interest, and I felt this fit well with that. I enjoy seeing a bit of development and wondering, “Where is this going?”

Insurance physician, female

Engagement was also influenced by “social proof and personalized recruitment.” Professionals reported that they were more inclined to join the HCD process after a colleague had personally recommended participation or confirmed that the process was worthwhile. This collegial endorsement provided reassurance and fostered trust, thereby encouraging participation.

While many professionals expressed motivation to contribute, engagement was also shaped by “uncertainties regarding the commercial character of the design sessions.” This ambiguity created hesitancy for some, who questioned the intentions of the organizing party and whether their input aligned with professional values or scientific interests. One participant stated:

Well, I do not know that company [organizing the design session], so why would I help this company with its commercial product? Whereas if it were different—let’s say you wanted to develop this product from an academic perspective—then I probably would have said, “Oh, sure, I will help, you know?” But that makes it different for me. If you know that research is involved, or that we are going to look at whether this actually has an effect in an evidence-based way, then I find that more valuable to contribute to than just helping another commercial company put its product on the market.

Occupational physician, female

### Elicit: Health Care Professional Input and Interaction During the Design Session

Health care professionals described their roles during the design sessions as “being a brainstormer” on the topic, appreciating the open space and using the opportunity to think aloud and provide relevant feedback and input for the platform’s development. They experienced the sessions as a space to “share experiences and bottlenecks from practice,” with the expectation that these insights would help shape a collaborative care network platform that is better aligned with the complexities of work-focused health care. As one participant explained:

Well, I hoped, so to speak, that I was able to provide a lot of information. A lot of information that can actually be used, something that is useful. That was my own idea of it—just share as much as possible, examples from practice. So that the people working on a problem, to come to a solution, can use all that information to reach a resolution.

Insurance physician private, female

Some professionals noted that “minimal presession preparation” lowered the threshold for participation and made it easier to join the session. Looking ahead, several professionals stated that, once the platform becomes more concrete, they could see themselves “sharing the platform’s existence” among colleagues, especially if the platform evolves into a usable form. One professional remarked:

I would briefly talk about [the platform], particularly with colleagues who are more involved in the IT side and data exchange. I would definitely mention it to them. [The platform] is already a bit more concrete than it was before. But I think that as I attend more sessions in the future, the more concrete it becomes, the more I will have to share -that is what I mean....Because for now, I would probably only say that this is something being worked on but that it is still in the early stages.”

Insurance physician, female

Alongside positive experiences, participants also noted barriers affecting interaction during the design session. Some professionals reported that it was “unclear what the platform’s purpose and personal relevance is” and that they received “limited information on the aim of the design session,” making it challenging to provide focused input. Several professionals indicated that a “defensive response by the session moderator” obstructed open dialogue and made them feel that their input was not taken seriously. Others reported “limited interaction during the design session,” resulting in one-sided exchanges. As one professional reflected:

Well, I think I mainly gave my input somewhat freely and did not give them much opportunity to speak, or ask many questions, so to speak. The time was up so quickly, and afterwards, I thought: Oh dear, I mostly just shared my own perspective. That was it. There might have been too little of a real conversation, you know—a bit one-sided, perhaps.

Clinical psychologist, female

Some professionals expressed that they could provide only “limited input due to a perceived lack of professional expertise,” as they did not see themselves as experts in medical information exchange or the development of digital tools. Differences in session pacing also influenced active interaction and input: some participants found the “duration of the session too short,” while others felt that the “duration of the session was long enough.” Practical barriers were frequently related to the virtual format. Several professionals described “challenges of a virtual design session,” such as delayed turn-taking or audio issues, as one professional expressed:

The only thing that was perhaps a bit less smooth was that, because it was on Teams, there were moments where you wonder, “Am I interrupting when I speak?” And there was one moment when I could not hear him properly. Those kinds of small things. But that was it—live, it might have been a bit easier.

Insurance physician, female

Additionally, presenting a “mockup platform as a conversational foundation to facilitate concrete understanding” helped guide professionals through the session. One professional noted:

I think it is the combination [of presenting a static mockup and interviewing]. The platform is the foundation, and the questions around it, which were clarified, make you provide answers. But the platform is definitely the foundation that guides the conversation.

Occupational physiotherapist, female

Professionals noted that, for the mock-up to be helpful, “good explanation while showing the design” was essential. Additional facilitators included “familiarity with similar system interfaces,” which made it easier to provide feedback, and the use of “clear and specific open questions,” which prompted more targeted input. Overall, many described the session as a “well-guided, structured discussion.” Several professionals also expressed that their contribution had an “impact due to involvement in human-centered design.” One professional shared:

Well, it definitely makes a difference, because you are involved, so you also feel like there is really an effort to listen to those who will actually work with it. What I often see in health care is managers, or other roles, who come in but are never on the work floor. They do not know how the process works, and then suddenly a process is rolled out that you are expected to implement. And we sometimes think, “Goodness, who came up with this? How is this even possible?” Not all steps are necessarily bad, but the process often hits a snag, and you think, “But this is a critical point where it fails.” You really need to involve the people who will actually work with it or, if it is for the work floor, those who will use it. That way, you know what you are doing with such a system and whether it is implementable for those who have to work with it. So, that is really nice. I truly believe that in this way, we can definitely contribute.

Medical specialist, cardiologist, female

Professionals also appreciated when the moderator (the UX developer of the platform) engaged directly in note-taking, describing this as “active user engagement,” which increased their sense of being taken seriously. Moreover, many reported experiencing a “safe environment for open and constructive feedback,” especially when explicitly invited to interrupt, comment, or raise concerns. One professional said:

Yes, he [the interviewer] offered that right at the start of the conversation: “You can interrupt me, or if you see something you want to comment on, feel free to do so.” And that is nice, because sometimes in these kinds of conversations, they only want questions one to ten answered, and then I might have something in my mind like, “Oh, but this does not look right, but I cannot say anything about it because that is not what the conversation is about.” But now, I could. It is then up to him, or the organisation, whether they do something with it, but at least I was able to express what my concerns or potential risks are.

Private insurance physician, female

### Elaborate: Reflection and Value of Participation

Health care professionals reported mixed expectations before participating in the design sessions. Some indicated that they had “no expectations prior to the design session,” while others anticipated a degree of interactivity and were “expecting a user interface mockup” based on previous experiences with digital design activities. As one professional explained:

I did indeed expect that we would navigate through some kind of product. I participated in something similar once before, which involved navigating a website where I had to search for items and see if I could find them. So, I thought maybe I would get assignments or something. Or they [the facilitator and designer] would show me something, to see if it looked good and if it was practical.

General practitioner, female

Professionals acknowledged the potential added value of participation and expressed a “positive experience with user involvement,” although some hesitated due to a “perceived unfavorable time trade-off” relative to their patient care responsibilities, offering them limited personal benefit. The “importance of designs” was recognized, with participants noting that even simple versions can reveal issues before further development. Furthermore, the “value of various backgrounds in co-creation” was highlighted, as professionals appreciated multiperspective engagement and the opportunity to hear diverse input, which they felt enriched the feedback process. For some, participation also contributed to personal learning. They described the session as providing “gaining more insight into rules and regulations,” particularly regarding data protection. Others mentioned “insight into progression in new developments,” expressing enthusiasm about moving from discussions on improving collaborative work-focused health care to actual implementation. One professional said:

For me personally, what was the added value: Well, I enjoyed it because I was kind of triggered with ideas. So, it also gave me a broader perspective on this issue, so to speak, on what it is for. And maybe I did not have such a clear picture myself of this particular solution direction, for example.

Private insurance physician, female

Several professionals highlighted that their participation in the session provided a glimpse into the future of more collaborative work-focused health care and helped them understand the platform’s potential value.

### Evaluate: Recommendations for Future End-User Involvement

Health care professionals provided multiple recommendations for future end-user involvement in the development of the communication platform. They emphasized the “need for a clear explanation of the platform’s broader aim in advance” and requested “clear and concise communication beforehand” regarding the platform’s goals. As one professional remarked:

When you are explaining something more complex or outside of what people already know, yes, then you really need to inform them beforehand. Otherwise, I would have needed the entire hour just to understand it, without even being able to give my reaction.

Private insurance physician, female

Others pointed to a “need for a clearer explanation of the development process,” particularly regarding the co-creation concept, which was unfamiliar to some professionals. To improve session effectiveness, participants recommended a “clearer introduction during the design session” and “enhanced participant preparation,” for example, through materials or examples provided in advance. One professional described the challenge of providing targeted feedback without sufficient prior context or familiarity. One professional said:

For us, as the audience, it is the first time....Sometimes it can catch you off guard a bit, putting you in a different mindset, like, “Whoa, hold on.” But without me meaning that negatively. For the person presenting, they are already familiar with it, but for us, it is new. So, take that into account—think about how you can make us a kind of co-owner of what is being presented. Because that will make you look at it differently. It might also make it easier to come up with suggestions for improvement—I do not want to call it criticism, but suggestions for improvement or changes—if you are more engaged with it.

Insurance physician, female

Professionals recommended including “illustrative examples,” such as user stories showing how a patient or professional would move through the platform, and offering a “demo for pretesting the platform” to help end users quickly understand its functionality. Some mentioned the value of “being able to autonomously navigate through the prototype platform,” even if it meant using a simple clickable mock-up. Others expressed a “need for feedback on their input,” seeking reassurance that their time investment had an impact and that their contributions were valued. To improve the session, professionals also mentioned a “need for reflection time,” noting that insights often emerged only as the session progressed or afterward. One professional noted:

But then I think the improvement point, if I may call it that, is that there should perhaps be an option to revisit it afterwards, to take the time to reflect calmly on the whole process. I also noticed during the conversation itself that as we progressed, I started thinking, “Oh, that is important too, and so is that, and that as well.” So, you kind of need to get into it, as it were.

Occupational physician, male

To enhance diversity and foster cross-pollination of ideas and input in the design process, professionals suggested “inviting a broader range of participants, including those without prior affinity for the topic,” and expressed a “desire for discussion with other professionals.” Finally, many professionals emphasized the value of moving toward “co-creation in multidisciplinary groups,” viewing cross-professional dialogue as essential for advancing and refining the platform in future development stages. As one participant described:

And I think it is very important to do this with more people. So, involve more professionals from different perspectives. It is not a problem at all if they come from different environments. Include a cardiologist, an occupational physician, an insurance physician, a general practitioner. Maybe even a client, if you can manage that. Then sit together, have the conversation, and look at how this can help us and what could be different or better....Because you also stimulate each other at some point. In those discussions, in those conversations, a dynamic emerges where you encourage and challenge each other to think beyond boundaries and share broader experiences. And you sometimes need that input to move forward.

Insurance physician, female

## Discussion

### Principal Findings

This mixed method study explored how health care professionals experienced their involvement in the development of a digital platform for more collaborative, work-focused health care. Using the 4 E’s of HCD participation framework (engage, elicit, elaborate, and evaluate), we gained insight into professionals’ motivations, roles, reflections, and recommendations for participating in design sessions. The findings show that, while professionals were generally motivated to contribute, their level of engagement and perceived value of the session were strongly influenced by how the process was introduced, facilitated, and followed up.

A key finding was that motivation to participate was often tied to a personal interest in the topic or innovation, a sense of professional responsibility, or social encouragement from colleagues. Previous research has examined the involvement of end users and distinguished between intrinsic motivation, where individuals engage in design processes because they find them personally meaningful or aligned with their professional identity, and extrinsic motivation, which is driven by external rewards or expectations [20]. In HCD in health care, intrinsic motivation has been shown to contribute to deeper engagement, stronger ownership, and more context-relevant input from professionals [14]. The distinction between intrinsic and extrinsic motivation for engagement is in line with our findings that professionals were most engaged when they saw a clear link between their professional expertise and the development goals of the platform. In our study, health care professionals expressed frustration when the purpose or ownership remained unclear. This was also highlighted in a previous narrative review, which showed that transparency about the design context and expected outcomes is essential to sustain trust and active contribution in HCD processes [14]. Additionally, our findings indicate that the perceived intentions and background of the organizing party (eg, commercial company vs academic institution) can influence professionals’ willingness to participate. Some participants expressed hesitancy when the design process was led by a commercial organization, associating academic or public initiatives more strongly with the public good and evidence-based development. However, literature suggests that commercial organizations can also be committed to public benefit and rigorous, human-centered innovation, especially when transparency and shared values are emphasized [8,17]. In terms of interaction, health care professionals in our study described their role during the sessions as “brainstormers,” suggesting that a generally open and safe environment for sharing ideas was created. This reflects a key principle in HCD: enabling participants to contribute freely [11]. However, design researchers in health care often face challenges in managing a dual role of both enabling open dialogue and directing discussions toward actionable outcomes [21]. This is also supported by our findings, where professionals appreciated the opportunity to share their ideas but also expressed a need for clearer preparation and guidance, particularly before the session. HCD offers a wide range of techniques to prepare and guide professionals, such as the use of sensitizing tools to prime participants and enhance engagement by encouraging reflection before an interview session [11,22]. Previous research argues that generative tools not only contribute to creativity but also empower participants to contribute more meaningfully [23]. In our study, professionals expressed the need for more context and clearer expectations before the session, which could potentially be addressed by such tools. Notably, some participants indicated that they were still unclear about the overall purpose and direction of the platform during the design sessions, describing the project as being “in the early stages” and difficult to communicate concretely to colleagues. This finding is concerning, as a core principle of HCD is to involve end users meaningfully from the outset, with clear communication about the goals and intended impact of the intervention. Lack of clarity at this stage may limit the depth and relevance of user input and could undermine engagement and ownership. This highlights the importance of providing participants with sufficient background information, context, and a clear articulation of the project’s aims before and during co-creation activities. Approaches such as shadowing have been shown to layer engagement strategies, allowing researchers to integrate various perspectives and better tailor design interventions to real-world complexity [24]. Applying similar preparatory methods in our setting could have helped balance the openness of idea sharing with more structured, context-informed discussion.

In addition, practical aspects such as the short duration of 1-hour sessions and the limitations of virtual interaction via video calls influenced professionals’ ability to contribute fully. This aligns with earlier findings that design researchers often face time constraints and infrastructural limitations, which can restrict the depth of user engagement [21]. To improve future co-creation efforts in digital health innovation, especially within complex environments such as cross-domain care settings, researchers and developers should consider allocating more time and resources [2]. Alternatively, using preparatory tools such as informational or instructional videos can help clarify key concepts and expectations in advance, allowing the actual session to remain concise while maximizing efficiency and user input.

The experiences of professionals also revealed a desire for more iterative involvement. Professionals expressed that they felt their participation was limited to a single design consultation, rather than being part of an ongoing development process. This underlines the importance of designing HCD processes that not only collect feedback on a single occasion but also show how user input informs development and offer opportunities for continued engagement. Iterative participation enhances a sense of co-ownership and contributes to better alignment between user needs and final interventions [11]. A central strength of HCD is that it directly involves those who will ultimately use or implement the intervention, ensuring that their practical knowledge and needs shape the outcome [11]. This was strongly reflected in our findings, as participants emphasized that active engagement in the design process increased their sense of being heard and contributed to the development of interventions that are more likely to be practical, relevant, and accepted in daily practice. Such involvement stands in contrast to top-down approaches, which often result in solutions that are less attuned to real-world workflows and may face resistance during implementation [25].

A core value of co-creation is the inclusion of diverse, interdisciplinary perspectives to enrich the design process and stimulate creative solutions. This was strongly reflected in our findings, as participants emphasized the importance of involving a broad range of professionals, and even patients, in collaborative sessions. This perspective is well supported in the literature, which shows that interdisciplinary co-design sessions foster creativity, mutual learning, and more contextually relevant and implementable interventions [11]. Interprofessional collaboration and the inclusion of diverse stakeholders, including patients, are recognized as best practices in HCD, as they help ensure that interventions are robust, acceptable, and sustainable in real-world settings [26]. While our findings reaffirm core HCD principles, such as the importance of user engagement and iterative feedback, this study uniquely highlights how the context of cross-domain health care and the nature of session facilitation (commercial vs academic) influence participant motivation and the effectiveness of co-design. These insights extend the current literature by illustrating practical challenges and opportunities for HCD in complex, real-world health care settings.

### Limitations

Although this study offers valuable insights into health care professionals’ experiences with HCD in the context of work-focused health care, several limitations should be acknowledged. First, the sample of health care professionals consisted mainly, though not exclusively, of individuals already interested in integrated care or digital innovation. This may limit the transferability and representativeness of the findings to less engaged individuals or those who are hesitant about digital interventions. Second, all design sessions were conducted online. While virtual formats offer convenience and accessibility, they may reduce the depth of interaction by limiting nonverbal communication. Third, participants were involved in a single design session during the design phase. A more continuous and iterative approach, with repeated engagement across phases, could have provided deeper insights into how professionals’ perceptions evolve and how feedback is integrated into the final product over time. Fourth, the authors were not involved in the design or facilitation of the design sessions. While this independent position reduced the risk of bias in our observations and analysis, it may have limited our ability to fully capture the rationale behind certain design choices or to assess the nuances of the facilitation process. Future studies may benefit from closer collaboration between evaluators and designers or from including multiple perspectives in the evaluation process.

### Implications for Practice

The findings of this study highlight the importance of a carefully structured and transparently communicated HCD approach, particularly in complex, cross-domain care settings. Developers of digital care platforms should invest in clear engagement strategies, such as using short videos or visuals to explain the goal, scope, and co-ownership of the platform. Sensitizing tools, such as brief presession assignments or opportunities for reflection, may further enhance user engagement. Facilitators of design sessions should carefully balance fostering open dialogue with providing structured guidance to ensure that the input gathered is both meaningful and actionable. Furthermore, timely follow-up communication that informs professionals involved in the design phase how their input has been incorporated can strengthen engagement, foster a sense of co-ownership, and ultimately contribute to more successful implementation. In addition, our findings highlight the importance of skilled facilitation in person-centered design sessions. Defensive or dismissive responses from session facilitators can undermine open dialogue and participant trust. Therefore, organizations leading co-creation or design sessions should invest in facilitator training focused on active listening, openness to feedback, and creating a safe, inclusive environment. Well-prepared facilitators are essential to ensure that all participants feel valued and that their contributions are genuinely considered in the design process.

### Implications for Research

This study adds to the literature on HCD in health care by applying the 4 E’s framework to examine the experiences of health care professionals as intended users of a digital platform in a real-world design phase. Our findings highlight the importance of aligning facilitation methods with the intended level of user participation. For rigorous person-centered design, it is essential to move beyond idea generation and feedback and to actively involve users in all stages of the design process through true co-creation. Future research could explore a more longitudinal and iterative participation approach and how this affects platform adoption. Additionally, perspectives from other stakeholders, such as patients, policy makers, and IT developers from other disciplines, would help build a more comprehensive understanding of suitable interventions in collaborative, work-focused health care.

### Conclusions

This study contributes to a deeper understanding of how to meaningfully involve health care professionals in the design of digital health innovations. Applying the 4 E’s of HCD participation revealed that motivation, quality of interaction, and iterative involvement are critical for effective co-creation, reinforcing and contributing to the existing body of literature on person-centered design in health care. To enhance future HCD efforts, clear communication, preparatory tools, and ongoing involvement should be prioritized, especially in complex, cross-domain care settings such as work-focused health care.

## Data Availability

The data that support the findings of this study are available on request from the corresponding author (NZ).

## Appendix

Multimedia Appendix 1 COREQ (Consolidated Criteria for Reporting Qualitative Research) checklist.

Multimedia Appendix 2 Observation checklist.

Multimedia Appendix 3 Interview guide.

## Abbreviations

COREQ: Consolidated Criteria for Reporting Qualitative Research
HCD: human-centered design
RTW: return-to-work
UX: user experience
