# Supplementary material for: Involving Health Care Professionals in the Human-Centered Design of a Digital Platform for Work-Focused Health Care: Lessons From a Mixed Methods Study
Source: JMIR Form Res. 2026 Apr 17;10:e83212. doi: 10.2196/83212 (PMC13135157; doi:10.2196/83212)
Supplement: Multimedia Appendix 2 [file formative_v10i1e83212_app2.docx]

**Supplementary Appendix 1**
**Observation Checklist: "Network Care for Work-Related Healthcare"**

**Objective**

The purpose of this observation checklist is to gather insights into the experiences of participants during the development process of the digital platform.

**Process**

1. **Session Introduction**
   - How was the session introduced?
   - What was the content of the introduction?
   - How long did the introduction last?
   - Was there room for questions?
   - Were the questions addressed?
   - Was the session's objective clearly explained?
2. **Questioning**
   - How were questions posed to the participant?
   - Was there sufficient room for questions from the participant?
3. **Role of the User Experience (UX) Designer**
   - What is the task of the UX designer?
4. **Session Conclusion**
   - How was the session concluded?
   - Was the follow-up explained to the participants?

**Participant Behavior**

- How does the participant respond to the proposed prototype?
- How is the communication between the UX designer and the participant?
- To what extent is the participant actively involved?

**Behavior of the UX Designer**

1. **Role**
   - What role does the UX designer play?
2. **Execution**
   - How does the UX designer fulfill this role?
3. **Communication**
   - How is the communication with the participant?

**Materials**

- Through which tool is the co-creation session conducted (e.g., Teams, Zoom, etc.)?
- What additional resources are used during the session?
- When are other tools or aids used?
- Why are these additional tools or aids used?
