# Supplementary material for: Involving Health Care Professionals in the Human-Centered Design of a Digital Platform for Work-Focused Health Care: Lessons From a Mixed Methods Study
Source: JMIR Form Res. 2026 Apr 17;10:e83212. doi: 10.2196/83212 (PMC13135157; doi:10.2196/83212)
Supplement: Multimedia Appendix 3 [file formative_v10i1e83212_app3.docx]

**Supplementary Appendix 2**
**Interview Guide: "Experiences with the Development of a Digital Platform"**

**Expectations**

1. What were your expectations prior to the co-creation session?
2. What motivated you to participate in the co-creation session?
3. To what extent were your expectations met?

**General Experience**

1. How did you experience the co-creation session for designing a digital platform?
2. What went well?
3. What could have been done differently?

**Role**

1. What was your role during the co-creation session?
2. How did you perceive your role during the session?
3. To what extent were you able to fulfill this role?

**Preparation**

1. To what extent were you informed about the purpose of the digital platform prior to the session?

**Materials**

1. How did you perceive the resources/tools used during the session?

**Communication and Interaction**

1. To what extent was there sufficient room for your questions regarding the co-creation process?
2. To what extent did you feel you could express and contribute your opinions?

**Future**

1. How do you see your role in the future regarding the digital platform?
2. Would you use the digital platform in the future?
3. Are there specific things you learned from the co-creation session?
4. What was the added value for you in participating in the co-creation session?

**Closing**

1. Is there anything else you would like to share regarding the development of the platform that we have not yet discussed?
